# The limits of medical recovery of post stroke patients suffering of different types of neoplasia

**DOI:** 10.1192/j.eurpsy.2021.1105

**Published:** 2021-08-13

**Authors:** I.D. Rădulescu, A.M. Pâslaru, V. Creangă-Zărnescu, A.-M. Fătu, A. Ciubară

**Affiliations:** 1 Psychiatrist, “Elisabeta Doamna” Psychiatric Hospital, Galati, Romania; 2 Corresponding Author, Phd Student, Faculty of Medicine and Pharmacy, University “Dunarea de Jos”, Galati, Romania; 3 Phd Student, Faculty of Medicine and Pharmacy, University “Dunarea de Jos”, Galati, Romania; 4 Md, Ph.d., Hab. Professor, Faculty of Medicine and Pharmacy, University “Dunarea de Jos” Head of Psychiatry Department, Senior Psychiatrist at ”Elisabeta Doamna” Hospital, Galati, Romania

**Keywords:** stroke, Neoplasia, recovery

## Abstract

**Introduction:**

A stroke represents a major cause of the disability of an adult with various biological, physiological and social implications. Excluding the characteristic neurological pathology, a series of complications may follow and if they are neglected they might compromise the success of medical rehab and the reintegration of the patient back into society.Recent studies have demonstrated that there is a higher rate of incidence of cancer among the survivors of a stroke in comparison with the general population.

**Objectives:**

The correlation between strokes and oncological disease.

**Methods:**

We have effectuated a prospective study of 6 months at the Neurology Section of Emergency Hospital “Saint Andrei”, Galati, in which we’ve included a total number of 50 patients who were over 60 years old. In this timeline we’ve analyzed the correlation between strokes and the comorbidities of the patient and the influence of these over the plan of medical rehab and the period of recovery after the stroke.

**Results:**

Over these 6 months, of all 468 patients having suffered strokes, 50 of these had been secondarily diagnosed with neurocognitive disorders. 56% of them were male and 44% were female, 37% from rural areas and 63% from the urban areas.

**Conclusions:**

The category of neurocognitive disorders includes the group of disorders in which the principal clinical deficit is located at the cognitive functions level and is usually acquired, not representing a disorder of development.

